# Corrigendum to: Small proline‐rich repeat protein 3 enhances the sensitivity of esophageal cancer cells in response to DNA damage‐induced apoptosis

**DOI:** 10.1002/1878-0261.13284

**Published:** 2022-07-20

The article by Luo et al. [1] contained inadvertent duplications between two western blot images presented in Figs 2B and 5A, and between two western blot images presented in Fig. 5B. The authors have corrected this by providing the original raw data for all experimental replicates, and the revised figures are included here. All authors agree to this corrigendum and confirm that changes do not affect the conclusions of the article.

The authors apologize for any inconvenience caused.

The corrected figures are reproduced below.

**Figure:**
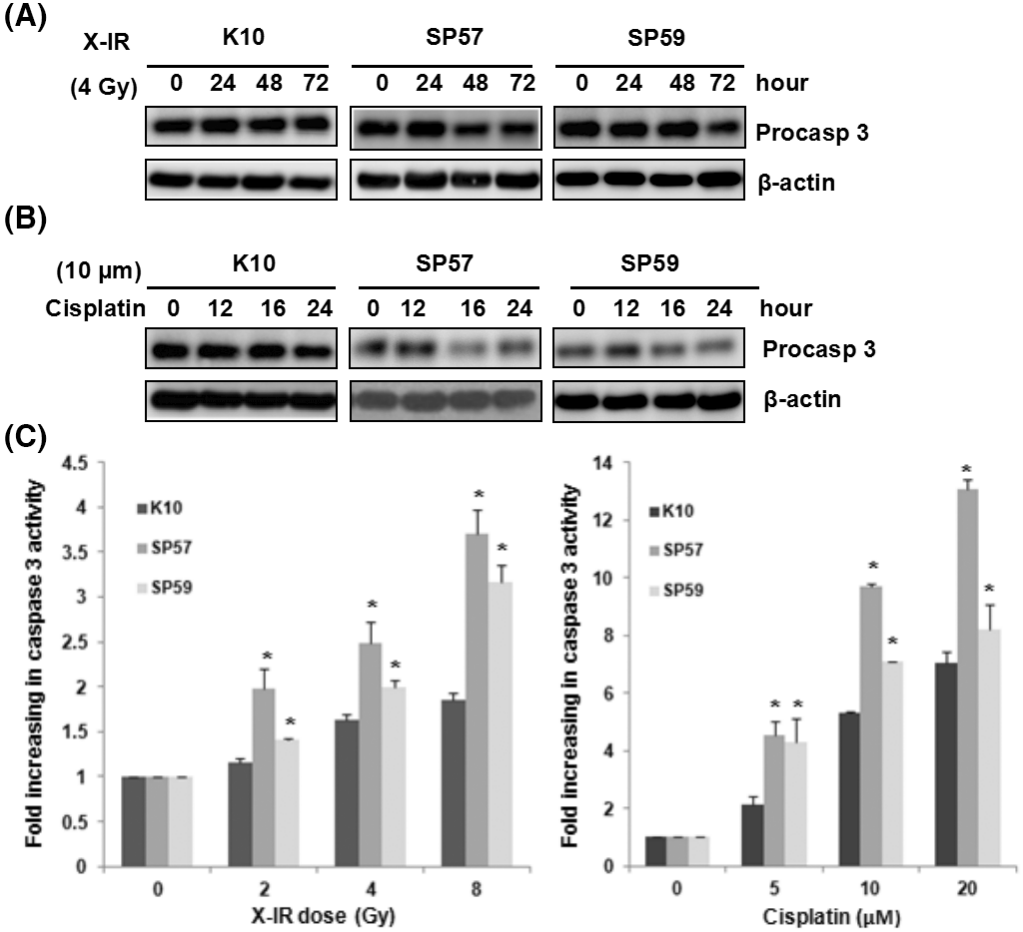
**Fig. 2.** Overexpression of SPRR3 triggered caspase activation in response to DNA damage. A & B) Overexpression of SPRR3 enhances the processing of caspase 3. Cells were exposed to cisplatin (10 mM, A) or X‐IR (4 Gy, B) and analyzed after the indicated time by Western blot for the processing of caspase 3. β‐actin was probed as a loading control. C) Cells were treated with cisplatin or X‐IR as indicated. Caspase 3 activity was measured by luminescent assay. Data shown are Mean ± SD from multiple independent experiments (*P < 0.05).

**Figure:**
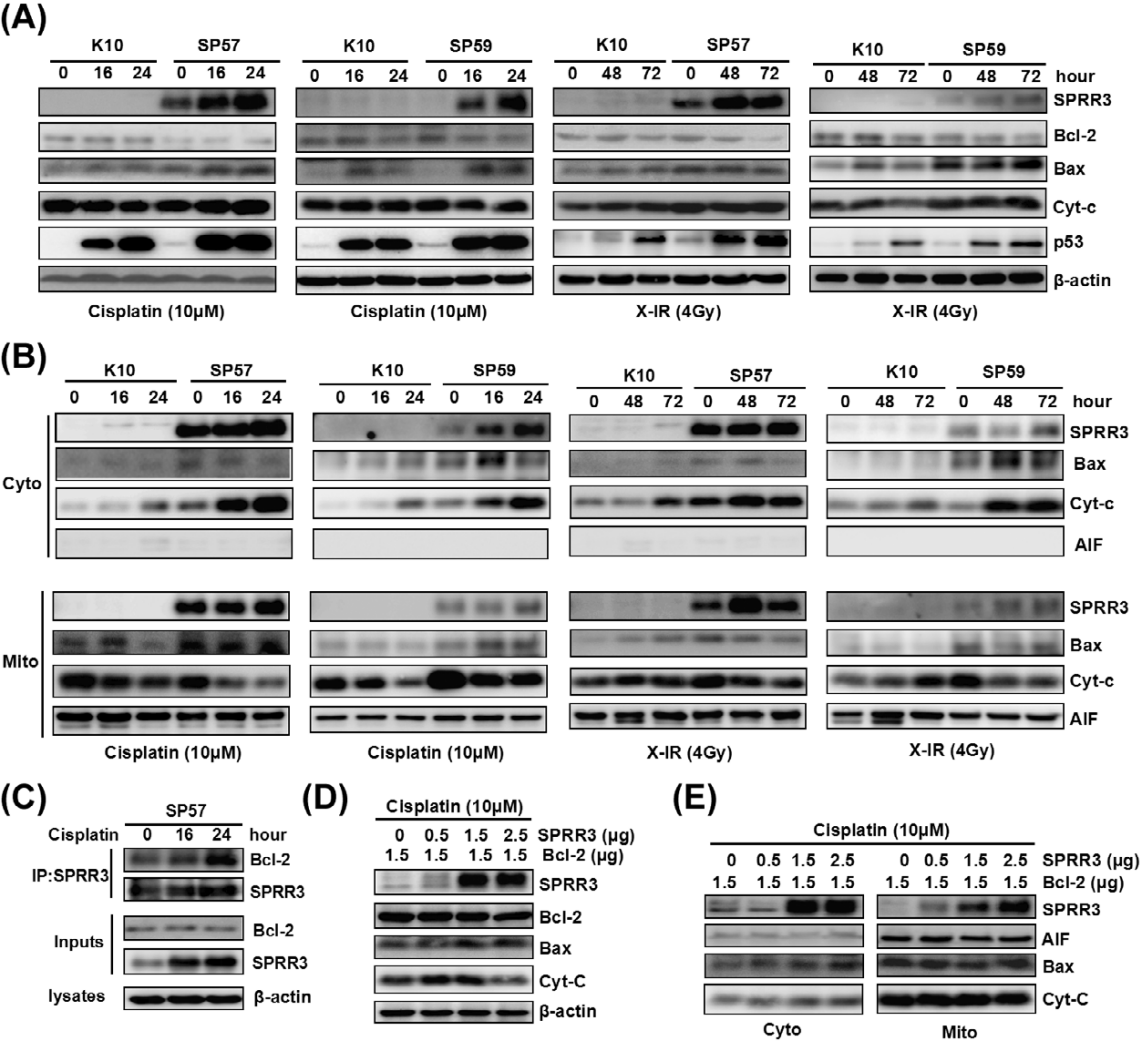
**Fig. 5.** Overexpression of SPRR3 activates Bax and causes the release of cytochrome c. A) Cells were exposed to cisplatin (10 mM) or X‐IR (4 Gy) at the indicated time. Overexpression of SPRR3 increased the expression of Bax and p53 compared with controls. B) Overexpression of SPRR3 caused Bax mitochondria translocation and the release of cytochrome c. Cells were subjected to subcellular fractionation after treatment. The cytosolic (Cyto) and mitochondrial (Mito) fractions were analyzed by Western blot. C) Co‐immnunoprecipitation analysis showing that interaction of SPRR3 with Bcl‐2 was increased after cisplatin treatment. D & E) Overexpression of SPRR3 abolished the Bcl‐2‐mediated anti‐apoptosis. EC9706 cells were transiently cotransfected with various amounts of HA‐SPRR3 with constant amount of GFP‐Bcl‐2 expression plasmids. After transfection, cells were treated with 10 mM cisplatin for 24 h and harvested. Cells were subjected to subcellular fractionation, and detected by Western blot.
